# Assessment of Residual Feed Intake and Its Relevant Measurements in Two Varieties of Japanese Quails (*Coturnixcoturnix japonica*) under High Environmental Temperature

**DOI:** 10.3390/ani9060299

**Published:** 2019-05-30

**Authors:** Moataz M. Fathi, Ibrahim Al-Homidan, Tarek A. Ebeid, Ahmed Galal, Osama K. Abou-Emera

**Affiliations:** 1 Department of Animal Production and Breeding, College of Agriculture and Veterinary Medicine, Qassim University, Al-Qassim 51452, Saudi Arabia; homidani@yahoo.com (I.A.-H.); tarkamin@gmail.com (T.A.E.); osama_emera@yahoo.com (O.K.A.-E.); 2 Department of Poultry Production, Faculty of Agriculture, Ain Shams University, HadayekShoubra 11241, Cairo, Egypt; galaly2k_2005@yahoo.com; 3 Department of Poultry Production, Faculty of Agriculture, Kafrelsheikh University, Kafr El-Sheikh 33516, Egypt; 4 Animal Production Research Institute, Agriculture Research Center, Dokki, Giza 12618, Egypt

**Keywords:** residual feed consumption, quail, high environmental temperature

## Abstract

**Simple Summary:**

Residual feed intake (RFI) is an important factor in improving poultry production and laying performance, particularly for poultry raised under heat stress. An experiment was conducted to assess RFI and its related measurements in Japanese quails (*Coturnixcoturnix japonica*) of two varieties (gray and white) reared under high environmental temperatures.The current results confirmed that raising gray quails for egg production under high environmental temperature is recommended. Multiple regression analysis clearly identified a significant effect of metabolic body weight and egg mass for the computation of expected feed intake rather than body weight gain in both varieties of Japanese quails.

**Abstract:**

Three hundred and ten 12-week-old laying Japanese quails (*Coturnixcoturnix japonica*) from gray and white varieties (155 each) were randomly selected from the initial population and kept in individual battery cages. The measurements of growth and egg production were determined to derive residual feed intake (RFI). The relationship between RFI and egg quality, blood parameters, and carcass characteristics was also determined. The results indicated that the gray quails had significantly higher egg mass and lower broken eggs compared to the white quails. A significant increase of eggshell strength and shell percentage was found in eggs produced from gray quails compared to their white counterparts, although their shell thickness means weresimilar. The results of multiple regression analysis clearly identified a significant effect of metabolic body weight and egg mass for the computation of expected feed intake, rather than body weight gain, in both varieties of Japanese quails. A strong positive correlation between RFI and feed intake in both gray and white quail varieties was found. The same trend was also observed for feed conversion ratio (FCR). Therefore, including RFI in the selection criteria of Japanese quails in order to improve FCR under high environmental temperature is highly recommended.

## 1. Introduction

Feed expenses represent almost 70% of the gross cost of poultry production. Lowering costs of maintenance processes would leave more energy remaining for higher output. Minimizing residual feed intake (RFI) and, in turn, improving feed efficiency would be beneficial for more efficient quail hens, particularly under high environmental temperatures. However, a bird’s ability to convert consumed feed to produce eggs and/or meat is greatly influenced by genotype and environmental factors. Birds that require less feed than expected for maintenance and production requirements have a negative RFI and are desirable in poultry breeding programs to reduce feed costs. RFI has increasingly become a critical factor for measuring feed efficiency and is commonly considered one of the target traits in animal breeding programs [1]. However, many selection programs take RFI into consideration to improve economic productive traits of synthetic or commercial strains. It has already been reported that RFI could be used in selection programs in laying hens and quails. Altan et al. [2] indicated that the selection for RFI in Japanese quails (*Coturnixcoturnix japonica*) might provide a tool to improve the efficiency of feed utilization without significant negative changes in egg production and egg quality traits and with a decreased susceptibility of the laying hens to stress. Most researchers concluded that a four-week recording period provides sufficient information for the genetic evaluation of residual feed intake in many species of poultry [3,4,5,6,7].

Direct selection for more efficient birds is becoming one of the primary goals in breeding programs of laying hens [7]. Improving feed efficiency is of great economic concern for commercial egg producers to maximize project outcomes. Traditionally, feed efficiency has been improved by selection for increased egg mass and decreased body weight and getting a correlated response in feed efficiency [2]. Identifying birds that require less feed for body maintenance could improve feed efficiency. However, selection for feed conversion ratio can lead to unfavorable changes in the component traits. Additionally, direct selection for feed efficiency requires measurement of individual feed intake, which is time consuming and very expensive. Well-designed feeders are also required to prevent feed wastage. On the other hand, additional criteria for feed utilization should be involved. Residual feed intake may be used as selection criteria to attain these goals [2]. To our knowledge, there are no previous reports on the residual feed intake of different lines or varieties of Japanese quails raised under hot ambient temperature [4]. Due to the need to adjust patterns of feed consumption according to ambient temperature, the present study was carried out to estimate RFI, as well as its relationship with productive traits, in two varieties of Japanese quails under high environmental temperature.

## 2. Materials and Methods

### 2.1. Birds, Housing, and Management

A total of 310 laying Japanese quails of two varieties (gray and white) (155 each) were randomly selected from an initial population of 800-day-old hens that were reared to point of lay and transferred to individual battery cages. Each hen was individually housed in a wire cage (20 × 20 × 20 cm) supplied with an individual feed trough in the front and a nipple drinker. The quail received a laying ration containing 18% crude protein (CP) and 2850 kcal/kg metabolizable energy (ME) during the experimental period. Throughout the experiment, feed and water were available ad libitum. Birds were exposed to a lighting period of 16 h per day.All quails received uniform care and management practices throughout the whole experimental period. The average high and low ambient temperatures recorded during the experimental period were 38.9 °C and 24.3 °C, respectively. No vaccination or medication was performed. The use and handling of quails were approved by the Ethical Committee of Qassim University.

### 2.2. Egg Production Parameters

Starting from 12weeks of age, egg production (weight and number), feed intake (FI), and body weight (initial and final) were determined for each hen over a four-week experimental period. Egg production (%) was calculated as total laid eggs divided by the total number of days (28 days). Feed intake was measured on a cage basis and combined with egg production data to calculate feed conversion ratio (FCR).

### 2.3. Egg Quality Measurements

A total of 460 eggs (230 eggs from each variety) were collected at 16 weeks of age. External and internal egg quality measurements were assessed according to Fathi et al. [8]. Each egg was weighed to the nearest 0.1 g. Egg width (equatorial axis) and egg length (longitudinal axis) were measured using Vernier caliper to 0.1 mm. Egg shape was calculated according to the following formula:

Egg shape = (egg width)/(egg length) × 100(1)

The breaking strength for intact eggs was determined in Kg/cm^2^ using Egg Force Reader^TM^, Orka Food Technology Ltd. (Wanchai, Hong Kong). The liquid contents were put aside and the shell plus membranes were washed under running water to remove the adherent albumen. The wet eggshell was left for 24 h at room temperature for drying and then weighed to the nearest 0.01 g. The relative weight of dry eggshell was calculated on the basis of egg weight. To measure shell thickness, pieces from three different regions (two poles and equator) of each eggshell with intact membranes were measured with a dial gauge micrometer to the nearest 0.01 mm. The height of thick albumen and egg yolk was measured by placing the liquid content on a balanced surface using a tripod micrometer. Then, the yolk was separated and rolled on tissue papers to remove the residual albumen. Albumen weight was calculated by subtracting the yolk and shell weight from egg weight. The weight of eggshell, yolk, and albumen were expressed as a percentage of egg weight. Haugh units (HU) were calculated according to the following formula:

HU = 100 log (H − 1.7W^0.37^ + 7.57) (2)

where H is the albumen height (mm) and W is the egg weight (g). Yolk color was measured by comparing yolk color to the DSM yolk color fan.

### 2.4. Carcass and Internal Organs

At the end of the experiment, 50 quails (25 from each variety) were randomly assigned for carcass yield assessment. After a pre-slaughter fasting period of 4 h, the quails were weighedand slaughtered by cutting their jugular veins. Following a 2-min bleeding time, each quail was dipped in a hot water bath at 60 °C for 60 s. and manually defeathered. Head and feet were removed. The carcass was eviscerated manually and weighed. Upon evisceration, the weight of eviscerated carcass, liver, heart, and gizzard was recorded and expressed as a percentage of live body weight according to Fathi et al. [9]. To minimize variations in the carcass procedure, all dissections were carried out by the same person.

### 2.5. Blood Hematology and Plasma Biochemistry

During slaughter, 25 blood samples were collected from each variety in heparinized tubes. The hematological parameters were determined by using Automatic Fully Digital Hematology Analyzer (BC-3000 Plus, Shenzhen Mindray, Bio-Medical Electronics Co., LTD, Shenzhen, China). These parameters were total count of red blood cells (RBC), hemoglobin (HGB), hematocrit (HT), and thrombocytes. The collected blood samples were centrifuged at 4000× rpm for 15 min. The resulting plasma samples were frozen at −20 °C for further analysis. The plasma concentrations of total protein, albumen, total cholesterol, and triglycerides were spectrophotometrically determined according to Fathi et al. [10] using commercial reagent kits (Stanbio Laboratory, Boerne, TX, USA). The globulin was calculated as the difference between the total protein and albumen.

### 2.6. Calculation of Residual Feed Intake and Statistical Analysis

Expected feed intake was computed using mid-metabolic body weight (BW^0.75^), body weight gain (ΔBW), and total egg mass (EM) for a given time considered by multiple regression analysis. Residual feed intake (RFI) was calculated as the difference between observed feed intake (OFI) and expected feed intake (EFI) for each experimental hen using the PROC REG procedure of JMP Ver. 11 (SAS, Cary, NC, USA) [11]. Each variety had its own partial regression coefficients according to the following multiple regression equation:

EFI = aBW_i_^0.75^ + bEM_i_ + cΔBW_i_ + d (3)

where EFI = expected feed intake of hen i (g); BW_i_^0.75^ = mean metabolic body weight of hen i (g^0.75^); EM_i_ = egg mass production of hen i (g); ΔBW_i_ = body weight gain (g); a, b, and c = partial regression coefficients; d = intercept.

Student *t* test analysis was applied to separate between means. All data were presented as means and the pooled SEM. Correlation coefficient was computed between RFI and some studied traits within each variety using PROC CORR procedure.

## 3. Results and Discussion

Productive performance of two varieties of Japanese quails is shown in Table 1. No significant difference for body weight (initial and final), weight gain, FI, or FCR was identified between the varieties. Gray quails had significantly higher (*p* < 0.02) egg mass and egg production percentage than that of white quails. A superiority of egg production in brown variety Japanese quails compared to both gray and white ones was previously detected [12]. Broken eggs were significantly (*p* < 0.05) affected by the quail variety. The gray quails recorded the lower value (1.13%) compared to the white quails (2.06%). Mortality levels fell within the normal range and there was no significant difference between quail varieties.

Internal and external egg quality characteristics are presented in Table 2. Shape index was significantly (*p* < 0.01) higher in eggs produced from white quails compared to their gray counterparts. Consistent with our results, Yilmaz et al. [13] and Sari et al. [14] reported that the egg shape index depended on the plumage color of the quails. They found that the mean shape index obtained from the gray plumage line was significantly lower than that of the white plumage line. In contrast to our results, Bagh et al. [10] did not find a significant difference between gray and white lines for all physical properties of egg quality. A numerical increase (*p* = 0.08) in HU was found in gray quails when compared with white quails. However, Bagh et al. [12] reported that there was no significance difference among the quail varieties for HU. In terms of yolk properties, there were no significant differences between quail varieties for yolk color, yolk index, and yolk percentage. Significantly (*p* < 0.04) higher albumen percentage was foundin eggs produced from white variety compared to the gray variety. In regard to eggshell quality, a significant (*p* < 0.01) increase in eggshell breaking strength was found in eggs produced from gray quails (1.43 kg/cm^2^) compared to the white quails (1.34 kg/cm^2^). Also, gray quails had a significantly (*p* < 0.01) higher relative weight of eggshell (9.4%) compared to that of their white counterparts (9.0%). However, shell thickness did not exhibit a significant difference due to the effect of variety. This advantage in eggshell strength associated with gray quails may be attributed to better ultrastructural featuresin comparison to eggshells of the white variety. Changes in external and internal quality characteristics of eggs obtained from quails with different plumage colors have previously been reported [12,13,14]. However, literature on the external and internal quality characteristics of eggs obtained from quail varieties with different plumage color under high environmental conditions is very limited.

Plasma biochemical and hematological parameters of gray and white feathered Japanese quails are summarized in Table 3. No significant effect on the blood biochemical variables was detected due to variety, except for cholesterol level. White quails had a significant (*p* < 0.01) increase in cholesterol level (198.5 mg/dL) compared to the gray variety (152.5 mg/dL). In terms of blood hematology, the white feathered quails had significantly higher levels of RBC, HGB, and HT compared to the gray quails. Generally, hematological parameters fell within the normal range for quails [15]. These results indicate that different genotypes in the present study were of normal physiological status.

Results of carcass traits studied as affected by variety of quails are shown in Table 4. It was found that neither carcass percentage nor giblets (liver, heart, and gizzard) significantly differed due to variety effect. However, an insignificant increase (*p* = 0.15) in carcass % was found in white quails (63.3%) compared to their gray counterparts (62.4%). Similar to the present study, Charati and Esmailizadeh [16] found that genotype had no significant effect on carcass percentage in white and wild (gray) Japanese quails. In contrast, several previous studies reported that feather color had a significant effect on live weight and carcass characteristics in Japanese quails. The white feathered quails had less body weight than that of the wild-type [17,18,19]. Similarly, Vali et al. [20] found significant differences in two quail strains for carcass weight, carcass percentage, and the relative weight of breast and femur.

The results of the multiple regression analysis are listed in Table 5. Below the table, the prediction equations for the expected feed consumption for each variety have been provided. RFI is defined as the difference between the realized feed consumption and the expected feed consumption, which was estimated based on metabolic BW, body weight gain, and EM [3,21]. As shown, the partial regression coefficients for metabolic body weight and egg mass had significant effects in computing expected feed intake in both quail varieties. The intercept value also had a significant effect. On the other hand, body weight gain (∆ BWT) did not significantly affect the computation of RFI in either the gray variety (*p* = 0.08) or the white variety (*p* = 0.63). Estimates of regression coefficients in the models of gray and white quails were close. Similar to our findings, Badawe et al. [3] found thatthe prediction of feed intake and residual feed intakederived from multiple regression analysis was significantly affected by metabolic body weight and egg mass in laying chickens (*gallus gallus*).

Phenotypic correlations between RFI and some studied traits are presented in Table 6. A strong positive correlation between RFI and FI in gray and white quail varieties (0.89 and 0.91, respectively) was found. Notably, the correlation between RFI and FI in our study was much higher than those estimated in previous works on laying chickens [7,22,23]. The selection for low RFI could reduce FI without significant changes in EM [7]. FCR is widely used but not a suitable selection trait because of its complex correlations with growth and production traits [6,7]. As shown in Table 6, a significantly high correlation was recorded between RFI and FCR (0.55 and 0.49, respectively). These strong relationships have indicated that selection for negative RFI would genetically improve feed efficiency and reduce feed intake. These results are consistent with those of Zhang et al. [1], who found a high phenotypic correlation between RFI and FCR (0.55). Moreover, RFI was strongly correlated with FI (0.82) in a random population of Pekin duck (*anas paltyrhynchos*). Similarly, RFI was positively correlated with FI in laying hens of chickens [22,23,24] and Japanese quails [2]. It is worthy to note that there was a low or neglected correlation between RFI and both egg weight and egg production% for the quail varieties. A low correlation between RFI with body weight gain was found (close to zero) in both quail varieties. Our results are in accordance with the findings of Luiting and Urff [25] and Altan et al. [2], who also described that the phenotypic correlation of RFI with both egg mass and body weight was almost zero. Likewise, these results are in agreement with the findings of Varkoohi et al. [5], reflecting the fact that RFI is phenotypically independent of weight gain. On the other hand, phenotypic correlations between RFI and both blood parameters and carcass traits were found to be rather low and insignificant. No significant relationship was observed between RFI with live body weight and eviscerated carcass weight [26].

## 4. Conclusions

The current results indicate that the egg mass significantly increased in the gray variety of Japanese quails compared to the white variety. Additionally, gray quails had a significantly lower percentage of broken eggs. Color variations in Japanese quails should be considered when selecting for type of production. We recommended raising gray quails for egg production under high environmental temperature, while the white variety may be more suitable for meat type. Results derived from multiple regression analysis clearly identified a significant effect of metabolic body weight and egg mass in computing expected feed intake rather than body weight gain in both varieties of Japanese quails. Including residual feed intake in the selection criteria of Japanese quailsin order to improve feed conversion ratio under high environmental temperature is highly recommended.

## Figures and Tables

**Table 1:** Productive performance of two varieties of Japanese quails (*Coturnixcoturnix japonica*).

Parameter	Variety		SEM	*p*-Value
	Gray	White		
Initial body weight, g	203.9	201.1	1.72	0.41
Final body weight, g	213.8	211.5	1.74	0.51
Body weight gain, g	9.8	10.4	1.32	0.84
Egg mass, g	289.5 ^a^	277.8 ^b^	2.43	0.02
FI, g	719.9	722.6	3.75	0.87
FCR	2.49	2.60	0.07	0.11
Egg production, %	89.9 ^a^	86.4 ^b^	0.73	0.02
Broken eggs, %	1.13 ^b^	2.06 ^a^	0.02	0.05
Egg weight, g	11.5	11.4	0.023	0.93

*N* = 155 quails/ variety; FI: feed intake; FCR: feed conversion ratio; ^a,b^ mean values in a raw without a common superscript are significantly different.

**Table 2:** Internal and external egg quality of two varieties of Japanese quails.

Parameter	Variety		SEM	*p*-Value
	Gray	White		
Shape index, %	75.95 ^b^	76.90 ^a^	0.15	<0.01
HU	88.7	88.2	0.13	0.08
Yolk color score ^1^	6.96	6.98	0.03	0.70
Yolk index	47.67	47.31	0.12	0.15
Yolk, %	33.5	33.3	0.12	0.39
Albumen, %	57.1 ^b^	57.7 ^a^	0.14	0.04
Shell, %	9.4 ^a^	9.0 ^b^	0.05	<0.01
Shell thickness, µ	254.0	255.2	0.73	0.40
Breaking strength, kg/cm^2^	1.43 ^a^	1.34 ^b^	0.01	<0.01

*N* = 230 intact eggs/variety; HU: Haugh units; ^1^ DSM yolk color fan. ^a,b^ mean values in a raw without a common superscript are significantly different.

**Table 3:** Biochemical and hematological blood parameters of two varieties of Japanese quails.

Parameter	Variety		SEM	*p*-Value
	Gray	White		
Total Protein, g/dL	5.34	5.43	0.17	0.82
Albumin, g/dL	3.13	3.42	0.09	0.27
Globulin, g/dL	2.21	2.01	0.18	0.69
Cholesterol, mg/dL	152.50 ^b^	198.47 ^a^	8.98	<0.01
Triglyceride, mg/dL	121.75	120.67	4.69	0.93
RBC	3.28 ^b^	3.51 ^a^	0.05	0.02
HGB	19.36 ^b^	20.59 ^a^	0.25	<0.01
HT	44.37 ^b^	47.21 ^a^	0.63	0.02
Thrombocytes	15.84	15.83	0.85	0.99

*N* = 25 quails/variety; RBC: red blood cells; HGB: hemoglobin; HT: hematocrit; ^a,b^ mean values in a raw without a common superscript are significantly different.

**Table 4:** Carcass traits of two varieties of Japanese quails.

Trait	Variety		SEM	*p*-Value
	Gray	White		
Live body weight, g	223.9	223.5	3.18	0.93
Dressed carcass, %	62.4	63.3	0.30	0.15
Liver, %	2.02	2.12	0.08	0.51
Heart, %	1.20	1.14	0.02	0.19
Gizzard, %	1.66	1.56	0.04	0.13

*N* = 25 quails/variety.

**Table 5:** Partial regression coefficients for factors affecting expected feed intake of two varieties of Japanese quails.

Parameter Estimate	Partial Regression Coefficient		Prob.	
	Gray	White	Gray	White
Intercept	914.6 **	396.2 *	<0.001	0.02
∆ BWT	−0.96	−0.36	0.08	0.63
(BWT)^0.75^	5.21 *	11.84 **	0.04	<0.001
Egg mass (EM)	−1.64 **	−1.11 **	<0.001	<0.001

*N* = 155 individual records/variety, *p < 0.05, **p < 0.01. Prediction equations: Y = 914.6 − 0.96 ∆ BWT + 5.21 (BWT)^0.75^ − 1.64 EM (Gray variety), Y = 396.2 − 0.36 ∆ BWT + 11.84 (BWT)^0.75^ − 1.11 EM (White variety); where Y stands for expected feed consumption, ∆ BWT = body weight gain, (BWT)^0.75^ = metabolic body weight, EM = total egg mass.

**Table 6:** Phenotypic correlations between RFI and some studied traits in Japanese quails.

Trait	Phenotypic Correlation	
	Gray	White
Δ Body wt	−0.003	0.002
Feed intake	0.89 **	0.91 **
FCR	0.55 *	0.49 *
Prod%	−0.07	−0.05
Egg wt	0.11	0.18
Total protein	−0.21	0.22
Albumin	−0.38	0.38
Globulin	0.01	0.03
Cholesterol	0.23	−0.15
Triglyceride	0.51	0.20
Hemoglobin	−0.18	−0.40
Red blood cells	0.32	−0.49
Hematocrit	0.20	−0.50
Thrombocytes	−0.15	−0.14
Carcass	0.09	0.09
Liver	0.04	−0.22
Heart	−0.38	−0.03
Gizzard	0.09	−0.22

* *p* < 0.05, ** *p* < 0.01.
